# Development and psychometric assessment of a scale for evaluating healthcare professionals’ attitudes toward interprofessional education and collaboration in the United States: a cross-sectional study

**DOI:** 10.3352/jeehp.2025.22.32

**Published:** 2025-10-20

**Authors:** Michael Christopher Banks, Ryan Brock Mutcheson, Maedot Ariaya Haymete, Serkan Toy

**Affiliations:** 1 Department of Anesthesiology and Critical Care Medicine, Johns Hopkins University School of Medicine, Baltimore, MD, USA; 2 Department of Health Systems & Implementation Science, Virginia Tech Carilion School of Medicine, Roanoke, VA, USA; 3 Medical Education, Virginia Tech Carilion School of Medicine, Roanoke, VA, USA; 4 Departments of Basic Science Education and Health Systems & Implementation Science, Virginia Tech Carilion School of Medicine, Roanoke, VA, USA; The Catholic University of Korea, Korea

**Keywords:** Health occupations, Interprofessional education, Psychometrics, Statistical factor analysis, Surveys and questionnaires, United States

## Abstract

**Purpose:**

Interprofessional education (IPE) is increasingly recognized as critical to preparing health professionals for collaborative practice, yet rigorous assessment remains limited by a lack of psychometrically sound instruments. Building on a previously developed questionnaire for physicians, this study aimed to expand the scale to include allied health professionals and to evaluate whether the factor structure remained consistent across professions. We hypothesized that a similar factor structure would emerge from the combined dataset, thereby supporting the scale’s generalizability.

**Methods:**

This observational study included 930 healthcare professionals in the United States (379 physicians, 419 nurses, 76 pharmacists, and others) who completed a 35-item questionnaire addressing IPE competency domains. Data were collected between December 2019 and May 2020. Exploratory factor analysis was employed to examine the factor structure, followed by item response theory (IRT) analyses to assess item fit, reliability, and validity. Raw data are available upon request.

**Results:**

Factor analysis of 22 retained items confirmed a 5-factor solution: teamwork and communication, patient-centered care, roles and responsibilities, ethics and attitudes, and reflective practice, explaining 59% of the variance. Subscale reliabilities ranged from α=0.65 to 0.87. IRT analyses supported construct validity and measurement precision, while identifying areas for refinement in reflective practice.

**Conclusion:**

This study demonstrates that the scale is reliable, valid, and generalizable across diverse health professions. It provides a robust tool for assessing attitudes toward IPE, offering value for curriculum evaluation, institutional benchmarking, and future longitudinal research on professional identity formation and collaborative practice.

## Graphical abstract

[Fig f1-jeehp-22-32]

## Introduction

### Background/rationale

In 1999, the Institute of Medicine published “To err is human: building a safer health system,” noting that the fragmented healthcare delivery system in the United States contributes to the costly errors and unsafe conditions [1]. Interprofessional collaboration plays a crucial role in delivering cost-effective health care while also enhancing patient outcomes and satisfaction levels for both patients and healthcare practitioners [2]. Hence, fostering interprofessional collaboration has become a key strategy for improving healthcare practice and quality of care [3,4]. Consequently, healthcare organizations have demonstrated support for interprofessional education (IPE) and collaborative practice, equipping individuals to work together to deliver high-quality patient care.

In 2001, the Institute of Medicine (IOM) published “Crossing the quality chasm: a new health system for the 21st century,” which stated, “Health professionals should be educated to deliver patient-centered care as members of an interdisciplinary team” [5]. According to the World Health Organization (2010), IPE occurs when “students from 2 or more professions learn about, from, and with each other to enable effective collaboration and improve health outcomes.” To address the IOM’s recommendations, medical schools and hospitals have adopted Interprofessional Education (IPE) and Interprofessional Collaborative Practice (IPCP). However, there is no clear consensus yet on the impact of IPE/IPCP curricula on health outcomes and on healthcare providers’ attitudes toward Interprofessional Education and Collaboration.

While interprofessional collaboration has been promoted to improve the quality of patient care, and IPE curricula are increasingly being included in health profession schools [3,4,6], psychometrically tested tools to examine healthcare professionals’ attitudes toward IPE/IPCP are limited. Most current studies addressing IPE/IPCP employ various questionnaires [7], underscoring the importance of using psychometrically sound scales to evaluate such attitudes among healthcare professionals from diverse disciplines.

The Readiness for Interprofessional Learning Scale (RIPLS) is among the most widely used tools developed to assess students’ attitudes in healthcare professions. However, this scale was not built on a sound theoretical framework [8]. An item response theory (IRT) analysis of the RIPLS found that while the scale demonstrated overall reliability, its subscale reliabilities were weak [9]. Similarly, evaluation of the TeamSTEPPS Teamwork Attitudes Questionnaire (T-TAQ) [10] and the Convergence Attitude Probe (CAP), a RIPLS-based scale, using IRT analysis revealed limitations, including poor model fit and variable discrimination [11]. These findings highlight the need for more robust measures of attitudes toward IPE. Currently, no psychometrically evaluated tools objectively assess learner outcomes of IPE/IPCP across the domains of IPE identified by Thistlethwaite and Moran [12] for the WHO and the Interprofessional Education Collaborative Expert Panel [6].

### Objectives

A recently developed questionnaire [13] measures physicians’ attitudes toward IPE/IPC across domains defined by the WHO and IPEC expert panels [3,4,12]. This study extends that work by incorporating the perspectives of nurses, pharmacists, and allied health professionals to re-examine the factor structure and its alignment with these frameworks. We aim to develop a psychometrically sound tool to assess the attitudes of a diverse group of healthcare and allied health professionals toward IPE and collaboration.

## Methods

### Ethics statement

This study received exempt status from the local institutional review board (IRB00222471). Participation in the online questionnaire was voluntary, and no incentives were provided; completing the questionnaire was considered informed consent. All data were collected and stored securely using Qualtrics survey software (Qualtrics). No personally identifiable information was collected. All procedures complied with the Declaration of Helsinki and institutional policies.

### Study design

It was a cross-sectional study designed to develop and psychometrically evaluate a scale measuring healthcare professionals’ attitudes toward IPE and collaboration.

### Setting

The study was conducted at a medical school located on the East Coast of the United States.

### Participants

Eligible participants included physicians (faculty and trainees), nurses, pharmacists, respiratory therapists, physical and occupational therapists, and registered dietitians affiliated with the medical school and health system. An email distribution list was created using institutional directories, and potential participants were invited to complete the survey via email. Three reminder emails were sent during the data collection period (December 2019–May 2020).

### Variables

The primary outcome variable was attitudes toward IPE and collaboration, measured across the following hypothesized domains: teamwork and collaboration, roles and responsibilities, communication, reflection and learning, the patient, and ethics and attitudes. Secondary variables included participant profession, specialty, and level of training.

### Data sources/measurement

We used the Interprofessional Education and Collaboration Scale (available for educational and research use with citation), a questionnaire previously developed and validated among physicians [13]. In that study, 35 items were created based on the WHO’s 6 domains for interprofessional learning outcomes [12], and exploratory factor analysis (EFA) supported a refined 20-item, 5-domain version. For the present study, we re-administered the original 35-item version distributed across 6 domains (7 teamwork and collaboration; 5 roles and responsibilities; 6 communication; 5 reflection and learning; 6 patient-centered care; 6 ethics and attitudes).

Items were rated on a 5-point Likert scale (1=strongly disagree to 5=strongly agree). All items were positively phrased; higher scores indicated more positive attitudes toward IPE and collaboration. Items were presented in random order to reduce order effects.

### Bias

Potential sources of bias included self-selection bias, as participation was voluntary, and the distribution of included professions, with a larger proportion of respondents from nursing and medicine compared to allied health groups. However, this distribution is broadly consistent with the composition of healthcare professionals at most academic medical centers in the United States, where physicians and nurses typically represent the largest workforce group.

### Study size

A total of 930 respondents completed the survey. Due to listwise deletion, the EFA was conducted with N=915. Although no a priori power analysis was conducted, our sample (N=915) exceeds both the absolute minimum sample size recommended for EFA (N>200) and the common guideline of 5–10 participants per item, supporting its adequacy for factor extraction and the robustness of subsequent psychometric evaluations.

### Statistical analysis

An EFA was first conducted to identify the scale’s underlying factor structure. Principal axis factoring with varimax rotation was used to maximize simple structure and interpretability. In addition to varimax, an oblique solution (direct oblimin, δ=0) was examined, given the conceptual relatedness of domains. The oblimin results were substantively similar, with modest factor correlations (r=0.12–0.28). For ease of interpretation, varimax loadings are reported. Items with factor loadings below 0.40 or cross-loadings greater than 0.40 were removed. Based on the final EFA results, composite subscale scores were computed as the item means; higher scores indicate more favorable attitudes, with no items being reverse-scored. Internal consistency was assessed using Cronbach’s alpha. All statistical analyses were conducted using IBM SPSS Statistics for Mac ver. 25.0 (IBM Corp.), with statistical significance set at P<0.05.

To further evaluate the psychometric properties of the subscales, we applied 2 polytomous Rasch models using Winsteps Psychometric Software ver. 5.6.0 (Winsteps.com) [14]: the partial credit model (PCM) and the rating scale model (RSM). Both the PCM and RSM were applied to evaluate item fit. The PCM allows item-specific thresholds, while the RSM constrains thresholds to be equal across items, making it appropriate for instruments with uniform Likert-type response categories. Comparing both provided complementary validity evidence and informed model selection.

Missing data were minimal (<2% per item) and handled using listwise deletion, with cases excluded only from the relevant analyses.

## Results

### Participants

The combined dataset included 930 healthcare professionals (Dataset 1). In the initial study, 379 physicians (221 faculty, 158 trainees) responded to the survey. In the updated study, the survey was completed by 552 allied health professionals (419 nurses, 76 pharmacist/pharmacy technicians, 22 respiratory therapists, 22 physical/occupational therapists, 12 registered dietitians). See Table 1 for the demographics. Most participants were from medicine and medical subspecialties (333/930, 35.8%) and surgery and surgical subspecialties (297/930, 31.93%). A total of 8 main specialties were represented in the study.

### Main results

#### Factor analysis

We first examined the factorability [15]. All items correlated with at least one other item with a value greater than 0.30. Anti-image diagonal measures of sampling adequacy values exceeded 0.75, indicating sampling adequacy. The Kaiser-Meyer-Olkin value of 0.94 (above the 0.06 threshold), and Bartlett’s test of sphericity was significant (χ^2^ (595)=12,765.07, P<0.001), confirming suitability for EFA.

Responses to all 35 items were subjected to principal axis factoring with orthogonal varimax rotation. Initial extraction suggested a 6-factor solution, explaining 55% of the variance; however, the 6th factor contained only 2 items and was difficult to interpret. A 5-factor solution was therefore retained. This finding led to the staged removal of 13 items with primary factor loadings <0.4 and/or cross-loadings >0.4. EFA of the remaining 22 items produced a stable 5-factor solution explaining 59% of the variance (Table 2).

The factors were “teamwork and communication,” “patient-centered care,” “roles and responsibilities,” “ethics and attitudes,” and “reflective practice.” Composite factor scores were calculated as the mean of included items, with higher scores reflecting more positive attitudes (Table 3).

#### Item response theory

Following the EFA, which identified 5 factors with acceptable internal consistency (Cronbach’s α ranging from 0.65 to 0.87), we conducted an IRT analysis to examine item performance further. As shown in Table 4, both the PCM and RSM produced similar item fit statistics, with most infit and outfit mean square (MNSQ) values falling within the acceptable range of 0.7–1.3, supporting the scale’s integrity.

The “teamwork and communication” factor (α=0.87) demonstrated strong item fit and a range of item difficulties, effectively capturing variation in attitudes toward teamwork. Similarly, the “patient-centered care” (α=0.77) also showed good item fit and balanced difficulty values, indicating sensitivity across respondents. By contrast, the “reflective practice” factor (α=0.65) had lower internal consistency and a narrower range of item difficulties, with some items (e.g., item #22) showing weaker fit in the PCM. Follow-up reliability analyses indicated that removing any item, including item 22, further reduced the α values (range, 0.54–0.62), suggesting that all 4 items contributed positively to the construct. IRT analyses also showed acceptable fit statistics for all items (infit/outfit MNSQ values within 0.7–1.3), supporting their retention. While the subscale captured essential aspects of reflective behavior, its lower reliability highlights an area for refinement in future studies.

Item difficulty values further illustrate the range of endorsement. Lower difficulty items (e.g., item #3 in “teamwork and communication”) reflected widely accepted views, while higher difficulty items (e.g., item #19 in “reflective practice”) required stronger attitudes to be agreed with. The “roles and responsibilities” and “ethics and attitudes” subscales also demonstrated acceptable item fit and balanced difficulty values (Table 4). Overall, the IRT analysis supports the factor structure and provides additional validity evidence, while also identifying potential areas for improvement.

## Discussion

### Key results

This study provides additional validity evidence for this scale to assess healthcare professionals’ attitudes toward Interprofessional Education and Collaborative Practice. The current study extends previous work that included only physicians by incorporating a broader range of healthcare professionals, including nurses, pharmacists, and allied health personnel [13]. This inclusion ensures the scale’s relevance and applicability across various disciplines within the healthcare sector. The consistency of the factor structure between our initial study, focusing solely on physicians, and this expanded study highlights the robustness and generalizability of our scale across different healthcare professions.

### Interpretation

EFA revealed a 5-factor structure: teamwork and communication, patient-centered care, roles and responsibilities, ethics and attitudes, and reflection and learning. Removing items with low or cross-loadings strengthened the scale’s psychometric properties, ensuring each item contributed meaningfully to the assessment of attitudes toward IPE and collaboration.

The 5 factors align with competency domains identified by the World Health Organization [4] and the Interprofessional Education Collaborative Expert Panel [3], confirming their relevance from healthcare professionals’ perspectives. Their replication across physicians, nurses, pharmacists, and allied health professionals underscores the universality of these core aspects. The intertwining of communication and teamwork reflects the collaborative nature of healthcare. At the same time, the distinct emergence of patient-centered care highlights the centrality of patient outcomes and perspectives to the quality of patient care.

Together, EFA and IRT analyses provided complementary evidence of reliability and validity. Both the PCM and RSM fit the data; however, the RSM offers a more parsimonious solution with fewer parameters, stable estimates, and theoretical consistency across items. Although the dataset was sufficient for the more flexible PCM, the RSM better fits our aim of simplicity and parsimony in scale interpretation.

### Comparison with previous studies

Our findings extend prior work on instruments such as RIPLS, T-TAQ, and CAP, which have demonstrated limitations in reliability and construct validity when evaluated using IRT methods [8-11]. Unlike these tools, the present scale demonstrated a stable 5-factor structure consistent with the WHO and IPEC frameworks [3,4,12] and acceptable reliability across its subscales. A recent scoping review of 29 instruments measuring interprofessional collaboration concluded that most tools focused narrowly on physicians and nurses and underwent only limited psychometric testing [7]. The present study contributes to this literature by providing a psychometrically validated scale applied across multiple professions, addressing both the narrow scope and limited validation highlighted in that review.

### Limitations

This study has several limitations. First, the response rate and potential self-selection bias may have skewed the sample toward participants with more favorable attitudes toward IPE. Second, the single-center design limits generalizability. Multi-center studies are needed to explore institutional differences and to confirm and refine the factor structure through confirmatory factor analysis. Third, the “reflective practice” subscale demonstrated lower internal consistency (α=0.65) than the other domains. While this level is acceptable for exploratory validation, it highlights an area for refinement in future research. Significantly, all items contributed positively, and IRT analyses supported their retention, suggesting that the subscale remains conceptually valuable despite its modest reliability. Another limitation is that this study did not test whether attitudes vary systematically by demographic characteristics (e.g., gender, professional experience) or whether items function equivalently across subgroups. Future research should explore these issues to strengthen evidence for generalizability and fairness. Finally, because physicians and nurses comprised the largest groups, the perspectives of smaller allied health professions may have been underrepresented, potentially biasing the results in favor of the dominant groups.

### Generalizability

The consistency of findings across multiple professions suggests that the instrument has potential applicability in a wide range of settings. However, its generalizability may be strongest in academic medical centers with workforce distributions similar to those in our sample. Application in rural, community-based, or international settings remains to be tested.

### Suggestions

Future longitudinal studies should investigate whether this scale can effectively detect changes in attitudes among healthcare learners and professionals over time. Future research should also extend validation with multi-group confirmatory factor analysis and differential item functioning analyses to test equivalence across professional groups. Additionally, cross-cultural validation in diverse countries and practice contexts will be essential to determine international applicability.

### Conclusion

This study provides validity evidence for a 22-item, 5-factor scale to assess healthcare professionals’ attitudes toward IPE and collaboration. Replicating the initially identified factor structure in physicians across a broader sample that includes nurses, pharmacists, and allied health professionals supports the scale’s generalizability. IRT analyses confirmed construct validity and highlighted reflective practice as a domain for future refinement. This scale provides educators and researchers with a robust tool for evaluating IPE initiatives, benchmarking institutional culture, and informing program design to prepare health professionals for collaborative practice better.

## Figures and Tables

**Figure f1-jeehp-22-32:**
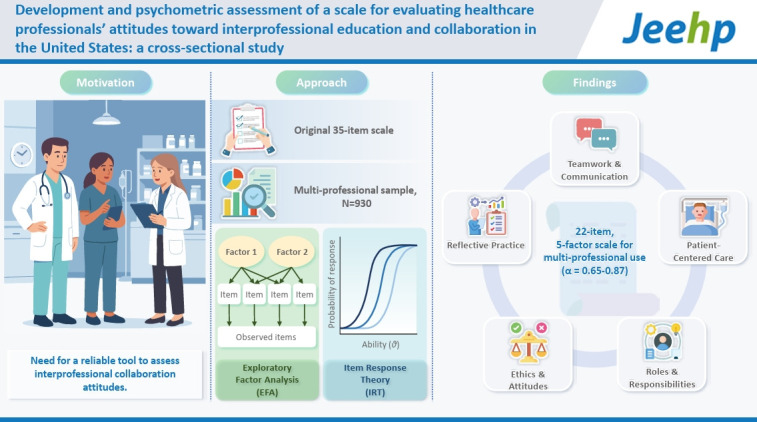


**Table 1.** Demographics of study participants (N=930)

Participant characteristics	Frequency (%)
Role/training level	
Nurse	419 (45.05)
Respiratory therapist	22 (2.37)
Pharmacist‎/pharmacy tech	76 (8.17)
Physical‎/occupational‎ therapist and SLP	22 (2.37)
Registered dietitian	12 (1.29)
Resident	158 (23.76)
Attending physician	221 (16.99)
Provider year of practice/training level	
PGY 1 and 2	47 (5.05)
PGY 3, 4, and 5	76 (8.17)
PGY 6, 7, and 8	34 (3.66)
0–2	116 (12.47)
3–5	128 (13.76)
6–10	137 (14.73)
11–15	109 (11.72)
≥16	282 (30.32)
Unknown	1 (0.11)
Medical specialty (N=930)	
Surgery and surgical subspecialties^a)^	297 (31.93)
Medicine and medical subspecialties^b)^	333 (35.80)
Pediatrics^c)^	123 (13.23)
Neurological sciences^d)^	41 (4.41)
Obstetrics and Gynecology	48 (5.16)
Psychiatry	32 (3.44)
Pathology	10 (1.08)
Radiologic sciences^e)^	18 (1.94)
Unknown	28 (3.01)

SLP, speech-language pathologist; PGY, post-graduate year.

a) Surgery and surgical subspecialties include: Surgery, Anesthesiology, Orthopedic Surgery, Ophthalmology, Urology, Plastic Surgery, Otolaryngology.

b) Medicine and medical subspecialties include: Internal Medicine, Medicine, Dermatology, Emergency Medicine, Oncology, Physical Medicine.

c) Pediatrics include: Pediatrics, Internal Medicine/Pediatrics.

d) Neurological sciences include: Neurological Surgery, Neurology.

e) Radiologic sciences included: Radiology, Radiation Oncology.

**Table 2.** Factor loadings based on a principal axis factoring with varimax rotation for 22 items from the updated Interprofessional Practice Scale (N=915)

Item	Teamwork communication	Patient centered care	Roles & responsibilities	Ethics & attitudes	Reflection & learning	Communality
1. Shared learning with other health care professionals will increase my ability to understand clinical problems.	0.762					0.54
2. Shared learning (training with other professionals) will help me become a more effective member of a health care team.	0.704					0.62
3. Shared learning helps to clarify the nature of patient problems.	0.682					0.56
4. Shared learning with other health care professionals will help me to communicate better with patients and other professionals.	0.660					0.66
5. I would welcome the opportunity to participate in training with other health care professionals.	0.627					0.41
6. Communication skills should be learned together with other healthcare professionals.	0.560					0.58
7. Establishing shared understanding helps optimize patient care.^a)^	0.516					0.46
8. I like to understand the patient’s side of the problem.		0.726				0.41
9. Thinking about the patient as a person is important in getting the treatment right.		0.686				0.57
10. Establishing trust with my patients is important to me.^a)^		0.512				0.59
11. The healthcare team should include the patient in the decision-making process.		0.505				0.35
12. I know the roles of other professionals in a healthcare team.			0.721			0.30
13. I am aware of the differences in scope of practice concerning team members from other disciplines.			0.699			0.43
14. I have a good sense of my professional role in a health care team.			0.558			0.38
15. Anyone in the health care team can make mistakes.				0.593		0.61
16. It is important for a team to function well under uncertainty.				0.539		0.44
17. All members of a health care team should respect each other.				0.470		0.55
18. I respect/value others’ opinions in the team no matter their role.				0.403		0.36
19. I research evidence-based practices related to team functioning to solve problems.					0.569	0.38
20. I reflect on team performance after events to plan future actions in a health care team.					0.546	0.37
21. I seek feedback from others about my role in a health care team.					0.492	0.39
22. I empathize with other professionals’ viewpoints in a team.					0.469	0.42
Eigenvalue	6.89	1.90	1.81	1.35	1.05	
% of total variance	31.32	8.66	8.23	6.14	4.76	-
Total variance (%)	59.11					

Factor loadings <0.40 are suppressed.

a) Indicates a new item added to the previously developed scale.

**Table 3.** Descriptive statistics for the Interprofessional Practice Scale (N=915)

Factor	No. of items	Mean±SD	Skewness	Kurtosis	Cronbach’s α
Teamwork and ccommunication	7	4.52±0.49	–1.02	1.05	0.87
Patient-centered care	4	4.67±0.41	–1.16	0.82	0.77
Roles and responsibilities	3	4.29±0.57	–0.70	0.80	0.74
Ethics and attitudes	4	4.67±0.37	–1.08	1.03	0.68
Reflective practice	4	3.22±0.63	–0.11	–0.27	0.65

SD, standard deviation.

**Table 4.** Item response theory analysis results: item measures and fit statistics

Construct	Item	Rating scale model					Partial credit model				
		Diff.	SE	Infit MNSQ	Outfit MNSQ	Pt Meas. Cor.	Diff.	SE	Infit MNSQ	Outfit MNSQ	Pt Meas. Cor.
Teamwork and communication	1	0.3	0.1	1.2	1.3	0.8	0.2	0.1	1.4	1.3	0.7
	2	0.0	0.1	1.1	1.2	0.7	–0.2	0.1	1.2	1.2	0.7
	3	–0.8	0.1	1.0	1.0	0.7	–0.4	0.1	1.1	1.0	0.7
	4	0.2	0.1	1.0	1.0	0.8	0.3	0.1	1.0	1.0	0.8
	5	–0.2	0.1	0.9	0.9	0.7	–0.8	0.1	1.0	0.9	0.7
	6	0.7	0.1	0.9	0.9	0.8	0.7	0.1	0.7	0.8	0.8
	7	–0.2	0.1	0.8	0.8	0.8	0.3	0.1	0.8	0.8	0.8
Patient-centered care	8	0.4	0.1	1.2	1.2	0.7	0.4	0.1	1.2	1.1	0.7
	9	–0.5	0.1	1.1	1.0	0.7	–0.6	0.1	1.1	1.0	0.7
	10	0.4	0.1	0.9	0.9	0.8	–0.4	0.1	1.0	0.8	0.8
	11	–0.2	0.1	0.8	0.7	0.8	0.6	0.1	0.8	0.8	0.8
Roles and responsibilities	12	–0.7	0.1	1.1	1.5	0.7	–1.6	0.1	1.3	1.5	0.7
	13	0.0	0.1	0.9	0.8	0.8	1.1	0.1	0.9	0.8	0.8
	14	0.7	0.1	0.9	0.8	0.8	0.6	0.1	0.8	0.7	0.8
Ethics and attitudes	15	–0.2	0.1	1.1	1.1	0.7	–0.0	0.1	1.1	1.1	0.7
	16	0.6	0.1	1.0	1.0	0.8	0.4	0.1	1.1	1.1	0.7
	17	–0.1	0.1	1.0	1.0	0.7	0.8	0.1	1.0	1.0	0.8
	18	–0.3	0.1	0.8	0.7	0.7	–1.2	0.1	0.8	0.8	0.7
Reflective practice	19	1.2	0.1	1.1	1.2	0.8	0.2	0.1	1.1	1.1	0.7
	20	0.3	0.1	0.9	1.0	0.7	1.3	0.0	1.0	1.0	0.8
	21	–0.5	0.1	0.9	0.9	0.7	–1.1	0.1	1.0	1.0	0.6
	22	–1.0	0.1	0.8	0.8	0.6	–0.4	0.1	0.9	0.9	0.7

Diff., difference; SE, standard error; MNSQ, mean square; Pt Meas. Cor., point-measure correlation.

## Footnotes

**Data availability**
Data files are available from https://doi.org/10.7910/DVN/FE5X1T Dataset 1. De-identified responses to the Interprofessional Education and Collaboration Questionnaire.

## Supplementary materials

Supplement 1. Audio recording of the abstract.
